# Ensuring Access to Immunoglobulin Therapies for People with Primary Immunodeficiency: A Need to Improve Individuals’ Quality of Life and the Sustainability of Health-care Systems

**DOI:** 10.3389/fimmu.2017.01165

**Published:** 2017-09-19

**Authors:** Ahmed Aziz Bousfiha, Carla Duff, Elena Hsieh

**Affiliations:** ^1^Clinical Immunology Unit, Casablanca Children Hospital CHU Averroes and Medical School King Hassan II University, Casablanca, Morocco; ^2^Division of Allergy and Immunology, Department of Paediatrics, University of South Florida, Tampa, FL, United States; ^3^Division of Allergy and Immunology, Department of Paediatrics, School of Medicine, University of Colorado, Children’s Hospital Colorado, Aurora, CO, United States; ^4^Department of Immunology and Microbiology, School of Medicine, University of Colorado, Children’s Hospital Colorado, Aurora, CO, United States

**Keywords:** primary immunodeficiency diseases, subcutaneous immunoglobulins, immunoglobulin replacement therapy, access to care

Classified as “rare diseases,” primary immunodeficiency diseases (PI) are hereditary and genetic disorders of the body’s immune system, which is partly or totally missing or does not function properly. These deficiencies lead to increased susceptibility to a wide range of infections affecting different parts of the body including the lungs, intestines, skin, ears, etc. and are often chronic, persistent, and debilitating. While antibody deficiencies are the most commonly diagnosed type of PI, over 300 forms exist (1), and because they often present themselves in the form of “common” infections, practitioners may just treat these infections while missing the underlying cause. This situation means infection can reoccur and leave the individual vulnerable to permanent organ damage, physical disability or even death. However, once recognized, these rare disorders are treatable and in some cases curable (2).

The seventh edition of the World Primary Immunodeficiency Week took place from the 22nd to the 29th of April 2017 with the aim to raise awareness of PI and ensure that the need for access to immunoglobulin (Ig) therapies for people with PI is recognized worldwide. Action needs to be taken to ensure effective and universal access to currently available treatment options for people living with PI, and particularly to the best-suited Ig replacement therapy as prescribed.

Primary immunodeficiency diseases are a universally accepted indication for Ig replacement therapy, which is a life-saving treatment for a majority of people with PI (3) as it offers protection against infections and reduces autoimmune symptoms (4). It consists of the regular administration of Ig therapies derived from human plasma providing antibodies that protect individuals against infections. In this sense, Ig therapies are “biological” medicines. Ig therapies have significantly improved the quality of life of people with PI, many of which can now live normal lives, and this is particularly important as antibody defects represent approximately half of the well-known PIs requiring Ig-replacement therapy (5). They have similarly decreased the frequency of infections and improved the prognosis of individuals.

It has been extensively demonstrated that early diagnosis and adequate implementation of appropriate treatment including Ig therapies are not only life-changing, life-enhancing for individuals, but also cost-saving for the health-care system (6), as they prevent the occurrence of unnecessary comorbidities and infections and thus represent significant decreases in the long-term cost of health care.

The success of Igs in antibody deficiencies administrated either intravenously or subcutaneously relies mainly on maintaining an adequate protection against infections. International guidelines recommend an Ig monthly dosage of 300–600 mg/kg body weight to be administered intravenously every 3 or 4 weeks or subcutaneously once/twice a week (7). Nevertheless, as the objective of Ig replacement therapy is to maintain one’s effective antibody level, treatment strategies shall be individualized and a personalized regimen (dosage and treatment route) must be developed for each patient, and modified as necessary to achieve treatment goals and meet the needs of each person, taking into consideration possible disease-associated complications. The importance of personalized treatment is all the more relevant given that many different Ig therapies are available, differing in terms of their ingredients and production, and individuals can respond differently to each of them.

However, barriers to an effective supply of the therapies to people with PI exist, and availability and access greatly vary across regions of the world but also across countries of the same region. Depending on geographical situation, the availability of Ig therapies ranges from none at all, to 15 products. Not all people with antibody deficiencies, therefore, have the chance to benefit from regular Ig treatment: an estimated 80% of people with PI do not have access to adequate care. Indeed, in some parts of the world, there is only a few to none patients diagnosed with PI, showing great disparities in access to diagnosis and care (8). On this point, the J Project and A Project have been created to raise awareness in Central Europe and Africa, respectively (9, 10).

There are several reasons for this situation, including the cost of the Ig therapies. In this context, the health-care system is a major determinant of patients’ health outcomes. Reimbursement on the national health-care system plays a key role in ensuring access to the therapies, but these are not always equally reimbursed in the different countries. As an example, treatment with intravenous or subcutaneous immunoglobulin G (IgG) is covered by the national health system in most European countries, but it is not consistently available in lower- or mid-income regions of the world. Reimbursement policies also vary between different Ig therapies and in some countries only one Ig product is reimbursed. In some cases, reimbursement is based on the type of PI, meaning that in the case of less severe PI, patients have to bear the cost of treatment on their own. Significant disparities in terms of reimbursement are also noticeable according to insurance plan options. Furthermore, in the light of a recent rise in the demand for plasma and the related need for increased numbers of donors, risks of global shortages have pressured access to the therapies, although the availability of Igs is an essential factor of successful treatment (3). The increasing demand for Ig has been driven by better recognition and diagnosis of antibody deficiencies, an aging population, the introduction of new therapeutic indications (11) and a lack of consensus on period treatment in some indications, for instance for some neurological illnesses, in addition to immune deficiencies (12), as well as the significant increase of secondary antibody deficiencies and, eventually, overprescription of Ig therapy to unindicated conditions by unexperienced clinicians. As scientific experts acknowledge that supply for people with PI must be given primary consideration as Ig treatments remain the main and only treatment option for most of them, this should be widely reflected in national clinical guidelines.

Addressing these inequalities would mean that in the future people with PI may have continuous, equal and optimized access to the widest range of safe and effective Ig therapies available, as supported by the Council of Europe’s resolution “on principles concerning human normal Ig therapies for immunodeficiency and other diseases” (13); and physicians may have the flexibility to choose the therapy tailored to the needs of each patient.

Stakeholders ranging from healthcare professionals, patient organizations, industry stakeholders to decision-makers and Governments need to work jointly to direct efforts toward this goal. While Igs are included in the World Health Organization List of Essential Medicines for both adults and children with PI, there are several areas for consideration by governments and regulatory authorities which include: appropriate supply of Igs on the national health-care systems to ensure equal access for all individuals and also guarantee (appropriate) reimbursement of the therapies; introduction of alternative funding mechanisms (subventions, health insurance) to ensure the availability of Ig products; evidence-based clinical guidelines for Ig use which recommend administration to people with PI and prioritize indications giving primary consideration to PI; and incentivize awareness-raising campaigns addressed to the general public for voluntary plasma and/or blood donations, as Ig supply is dependent upon plasma availability.

It is essential that steps are taken at all levels to fully support Ig treatment in PI and to consider such treatment a priority in maintaining optimal quality of care for people with PI.

**Table:** 

Take-home messages
Principal goal of Ig replacement therapy is to prevent infections/organ damage in patients with defects in antibody production/function. Intermittent or low dose IgG result in low serum levels and poor control of infections. Cost implications may prevent optimal dosing in developing countries. When studies have been done, Ig therapy has been found to be cost-effective in preventing hospitalizations/emergency visits, expensive antibiotic treatments and missed days of school/work and it is listed by WHO as essential medicine with no alternative. Ig therapy should be made available for selected PID patients in all developing countries as soon as they can be diagnosed and managed by trained physicians experienced in PID.

## Author Contributions

Each author contributed to the drafting of the paper by producing several paragraphs and editing the content of other sections. Please note that Bénédicte Faure is project manager of the World PI Week campaign and submitting the paper on behalf of the authors.

## Conflict of Interest Statement

The World Primary Immunodeficiency Week is financially supported by the pharmaceutical companies CSL Behring and Shire. Editorial control rests with the World PI Week Steering Committee members who author the publications.
